# Messenger RNA exchange between scions and rootstocks in grafted grapevines

**DOI:** 10.1186/s12870-015-0626-y

**Published:** 2015-10-19

**Authors:** Yingzhen Yang, Linyong Mao, Yingyos Jittayasothorn, Youngmin Kang, Chen Jiao, Zhangjun Fei, Gan-Yuan Zhong

**Affiliations:** United States Department of Agriculture, Agricultural Research Service, Grape Genetics Research Unit, Geneva, NY 14456 USA; Boyce Thompson Institute for Plant Research, Cornell University, Ithaca, NY 14853 USA; United States Department of Agriculture, Agricultural Research Service, Robert W. Holley Center for Agriculture and Health, Ithaca, NY 14853 USA; Present address: Department of Biochemistry & Molecular Biology, Howard University, 520 W Street, NW Washington, D. C, 20059 USA; Present address: Laboratory of Immunology, National Eye Institute, National Institutes of Health, Bethesda, MD 20892 USA; Present address: K-herb Research Center, Korea Institute of Oriental Medicine, Deajeon, 305-811 Republic of Korea

**Keywords:** mRNA trafficking, Detection of mobile mRNAs, Genome-wide, mRNA exchange, Diagnostic SNP, Transmission rate, Grapevine, Graft genetics

## Abstract

**Background:**

Grafting has been widely practiced for centuries in the propagation and production of many vegetable and fruit species. However, the underlying molecular and genetic mechanisms for how the graft partners interact with each other to produce a successful graft remain largely unknown. We hypothesized that genome-wide mRNA exchanges, which were recently documented in grafted model plant species, are a general phenomenon widely present in grafted plants, including those in vegetable and fruit species, and have specific genotype- and environment-dependent characteristics modulating plant performance.

**Methods:**

Using diagnostic SNPs derived from high throughput genome sequencing, we identified and characterized the patterns of genome-wide mRNA exchanges across graft junctions in grafted grapevines grown in the in vitro and field conditions.

**Results:**

We identified more than 3000 genes transporting mRNAs across graft junctions. These genes were involved in diverse biological processes and those involved in basic cellular, biosynthetic, catabolic, and metabolic activities, as well as responses to stress and signal transduction, were highly enriched. Field-grown mature grafts had much fewer genes transmitting mRNAs than the *in vitro* young grafts (987 vs. 2679). These mobile mRNAs could move directionally or bi-directionally between scions and rootstocks. The mRNA transmission rates of these genes were generally low, with 65 % or more having transmission rates lower than 0.01. Furthermore, genotypes, graft combinations and growth environments had impact on the directions of mRNA movement as well as the numbers and species of mRNAs being exchanged. Moreover, we found evidence for the presences of both passive and selective mechanisms underlying long distance mRNA trafficking in grafted grapevines.

**Conclusions:**

We extended the studies of mRNA exchanges in model species to grapevines and demonstrated that genomic-scale mRNA exchange across graft junctions occurred in grapevines in a passive or genotype and environment-dependent manner.

**Electronic supplementary material:**

The online version of this article (doi:10.1186/s12870-015-0626-y) contains supplementary material, which is available to authorized users.

## Background

A grafted plant is usually composed of two genetically distinct parts: scion and rootstock. The scion and rootstock are joined together through a graft junction forming a composite plant. Grafting is an ancient agricultural practice and has been widely used in the propagation and production of many vegetable and fruit species [1, 2]. Important grafting applications include using rootstocks for clonal propagation of scions with rooting difficulty, control of plant architecture, induction of precocious flowering, enhancement of disease and pest resistance and soil adaptation [1]. A well-known graft example in fruit species is the successful use of resistant wild American grape species as rootstocks for control of the devastating phylloxera disease in the widely cultivated European grape species *Vitis vinifera* [3].

In order for a composite plant to survive and grow successfully, a functional vasculature system of xylem and phloem needs to be established across the graft junction. While the xylem stream mainly transports water and other inorganic compounds driven by the pull of transpiration from root to shoot, the phloem stream carries organic nutrients from source to sink organs/tissues driven by the pressure gradient [4, 5]. It was long believed that only small molecules, such as water, hormone, ions, amino acids and photoassimilates, could be transported from source to sink tissues via the phloem system. However, some large molecules, such as proteins and RNAs, were detected in phloem sap [6, 7] and RNAs have been proposed to function as long distance signaling molecules [4]. RNA species have been detected from phloem exudates collected using various methods from different plant species [8–12]. Collectively, hundreds or even thousands of mRNA species have been identified from various phloem exudates. However, only a few of them exhibited long-distance physiological functions with most examples from transgenes or dominant mutants [13–22]. It was quite puzzling why there were so many mRNA species detected in phloem sap, but only few known cases of endogenous mRNAs were reported to have long distance functions [23, 24].

RNA molecules, especially mRNAs, often have large molecule weight [5]. Theoretically they would not be able to go through plasmodesmata to reach the phloem stream without assistance. It has been suggested that mRNA long distance movement is a selective process [11, 19] and certain RNA tertiary structures or elements are necessary for long distance RNA trafficking [15, 21, 25–27]. Additional studies suggested that certain ribonucleoproteins can bind to mRNAs specifically or non-specifically and mRNAs might move in a ribonucleoprotein complex [28, 29]. However, diffusive/nonspecific movement or mass flow in the phloem stream has also been suggested for long distance mRNA translocation [1, 5]. The perplexing fact that many mRNA species have been detected in phloem sap without apparent long distance functions supports that passive diffusion of mRNAs in the phloem stream likely takes place as well.

Recent studies revealed extensive mRNA exchange between *Arabidopsis* and its parasitic plant *Cuscuta pentagona* through symplastic junctions [30, 31], between inter-generic grafts of *Arabidopsis* and tobacco [32], and between intra-specific grafts of *Arabidopsis* through graft junctions [33]. However, these reports were based on model species and how the results from these studies can be applied to agricultural graft crops is unknown. In this study, we extended the studies of mRNA movement in model species to grapevines, a woody fruit species of significant economic importance, and provided insights into how genome-wide mRNA exchange between scions and rootstocks may contribute to the genetic success of grafted plants.

## Results

### mRNA exchange between scions and rootstocks

**Fig. 1:**
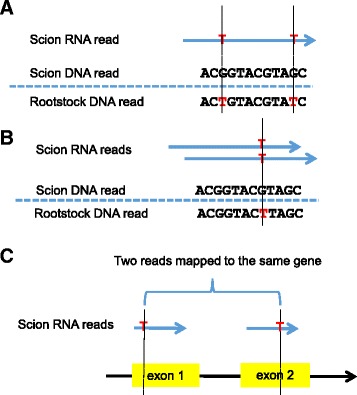
Detection of mobile mRNAs. Illustrated are examples for the three cases of mRNA movement detected in this study. The mobile mRNA transcripts in the scion (receptor) are perfectly aligned to the rootstock (donor) genome, and have (**a**) at least one read carrying two or more diagnostic SNP loci (colored “T”s); (**b**) at least two unique reads covering one diagnostic SNP locus; or (**c**) at least two unique reads carrying different diagnostic SNP loci

Two sets of grafted materials, one grown *in vitro* and the other in the field, were investigated in this study (Table 1 and Additional file 1: Figure S1). To detect mRNA exchange between scions and rootstocks, we mapped genomic sequencing reads of the scions and rootstocks to the *V. vinifera* reference genome, determined their genotypes, and identified diagnostic SNP loci between respective scions and rootstocks following our computational pipeline as described in the Material and Methods. Similarly, RNA-Seq reads from individual rootstocks and scions of various grafts were separately aligned to the *V. vinifera* reference genome. The transmission ability of a transcript was determined by comparison of corresponding genomic and RNA-Seq reads of rootstocks and scions. A transcript is defined as mobile if its corresponding RNA-Seq reads from the donor were detected in the receptor’s RNA-Seq library (Fig. 1 and Additional file 1: Figure S2). A gene which produces mobile transcripts between scion and rootstock is described thereafter as a graft transmitting gene.

**Table 1:** Graft genotypes and combinations, growing conditions, tissue sampling, and numbers of genes with mobile mRNA reads detected

Growing condition	Sampling time	Genotype		Scion			Rootstock			Total no. of genes	Average transmission rate
		Scion	Rootstock	Tissue sampled	Mapped Reads^a^	No. genes	Tissue sampled	Mapped Reads	No. genes		
*in vitro* ^b^	4 weeks after grafting	*V. girdiana*	*V. palmata*	shoot, leaf, stem	98.4 M	1130	shoot, leaf, stem, root	112.3 M	646	2679	0.0238
*in vitro*	4 weeks after grafting	*V. palmata*	*V. girdiana*	shoot, leaf, stem	104.9 M	1125	shoot, leaf, stem, root	100.5 M	747		
Field^c^, pH5.5^d^	11 years after grafting	*V. vinifera* cv. ‘Riesling’	*Vitis* hybrid ‘C3309’	young shoot	64.8 M	80	small root	79.7 M	555	987	0.042
Field, pH6.5^e^	11 years after grafting	*V. vinifera* cv. ‘Riesling’	*Vitis* hybrid ‘C3309’	young shoot	91.6 M	134	small root	66.4 M	517		

^a^The number of 101-bp RNA-Seq reads (in millions) mapped to the grape reference genome

^b^Each *in vitro* graft combination had three or more grafted plants which were bulked in tissue sampling

^c^The grafted plants were planted in the field in 2003. Tissues from six plants from each field condition were pooled as a bulk sample

^d^Soil was untreated

^e^Soil was treated with limestone to improve the soil pH level

About 95 to 175 million reads with length of 101 bp were produced for each individual RNA-Seq library and 56 to 68 % of these reads were mapped to the *V. vinifera* reference genome (Additional file 2: Dataset S1). Collectively, 3333 graft transmitting genes were identified from these two sets of grafted materials (Additional file 2: Dataset S2). Among them, 1188 genes had mobile transcripts detected in at least two different graft materials. The number of transmitting genes varied among different grafted materials. Mobile transcripts from 2679 genes were detected in the *in vitro* reciprocal grafts between *V. girdiana* and *V. palmata*. In contrast, 987 transmitting genes were detected in the field grafts with ‘Riesling’ as scions and ‘C3309’ as rootstocks (Table 1 and Additional file 2: Dataset S2).

Gene Ontology (GO) term analysis of the 3333 graft transmitting genes, using the Plant MetGenMAP analysis tool [34], indicated that diverse biological processes were over-represented including those related to many basic cellular, biosynthetic, catabolic, and metabolic activities, as well as responses to stress and signal transduction (Additional file 2: Dataset S3). GO term analysis also revealed a number of over-represented molecular functions, among which one was related to passive transmembrane transporter activity.

Transcription factors involved in development and hormone signaling are among the genes whose mRNAs were often found in plant phloem samples [8, 9, 15, 19, 21, 35–38] and some of which were confirmed grafting-transmissible, including *CmNACP*, *StBEL5*, a *Knotted 1*-like transcription factor, *GAI* and a few *Aux*/*IAA* genes [8, 11, 13, 14, 16, 21, 37, 39]. mRNAs for many of these genes were also found to be mobile in this study, including those coding for NAC-domain containing proteins, BEL1 homologs, Myb, WRKY, GATA, and knotted-1 like transcription factors (Additional file 2: Dataset S4). Furthermore, mobile transcripts were detected for many genes encoding proteins involved in the metabolic and signaling pathways of different plant hormones, including auxin, gibberellin, abscisic acid, ethylene and jasmonic acid (Additional file 2: Dataset S4). These results not only confirmed some of the previous observations (see the references in Additional file 2: Dataset S4), but also provided further evidence that these categories of genes in general were more likely to produce mobile mRNAs in grafted plants.

### mRNA movement in the reciprocal *in vitro* grafts

**Fig. 2:**
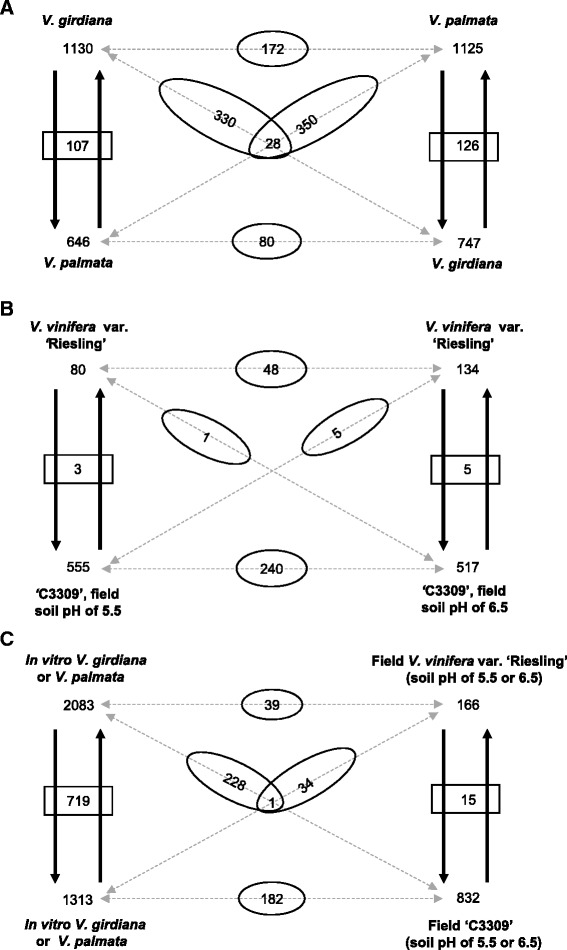
Diagrams of mRNA movement in the *in vitro* and field grafts. Up and down arrows and their pointing numbers respectively represent the moving directions and numbers of genes producing mRNAs moved into scions (up) or rootstocks (down). Numbers in rectangle boxes indicate the numbers of genes whose mRNAs moved in both directions. Numbers in ovals indicate the numbers of genes shared between the two groups connected through dotted lines. (**a**) mRNA movement in the *in vitro* reciprocal grafts. mRNAs from the 28 genes noted in the overlapped two ovals moved in both up and down directions and both genotypes. (**b**) mRNA movement in the field grafts. (**c**) Comparisons of mRNA movement in the *in vitro* and field grafts

We observed that 2679 genes transmitted mRNAs across graft junctions in the *in vitro* reciprocal grafts. Among them, 736 transmitted transcripts in both grafts (Additional file 2: Dataset S2). We further observed that more mRNA species moved into the scion tissues in both the reciprocal grafts, regardless of the scion genotypes. However, the numbers of mobile mRNAs produced and transmitted by *V. girdiana* and *V. palmata* were similar when they served as rootstocks or scions in these grafts (Fig. 2a). Specifically, in the graft of *V. girdiana* (scion)/*V. palmata* (rootstock), mobile mRNAs of 1130 *V. palmata* and 646 *V. girdiana* genes were respectively found in the scion and rootstock tissues. Between these two sets of transmitting genes, 107 were transmitted bi-directionally into both *V. girdiana* and *V. palmata*. Similarly, mobile mRNAs of 1125 *V. girdiana* and 747 *V. palmata* genes were respectively found in the scion and rootstock tissues of the *V. palmata* (scion)*/V. girdiana* (rootstock) graft and 126 of these genes had mobile mRNAs moved bi-directionally into both *V. girdiana* and *V. palmata* (Fig. 2a). We compared the mobile RNA species received by the two different scion genotypes of the reciprocal grafts and found 172 were in common. Likewise, mRNA species of 80 genes were found to move into both rootstocks. Between the set of the 1130 *V. palmata* genes transmitting mRNAs from the *V. palmata* rootstock to the *V. girdiana* scion and the set of 747 *V. palmata* genes transmitting mobile mRNAs from *V. palmata* scion to *V. girdiana* rootstock, 330 genes were overlapped. Similarly, 350 genes were overlapped between the two sets of *V. girdiana* transmitting genes when *V. girdiana* respectively served as rootstock and scion (Fig. 2a). There were 28 genes whose mRNA species moved into both graft partners in both reciprocal grafts (Fig. 2a and Additional file 2: Dataset S2).

We performed GO term analysis on the 2679 transmitting genes observed in the *in vitro* grafts, and found that among the over-represented biological processes were those related to responses to certain forms of stresses or stimuli, such as water, ions, and hormone, signal transduction, membrane organization, photosynthesis, biosynthesis, and various cellular metabolic activities (Additional file 2: Dataset S5). Over-representation of these diverse biological processes in the mRNA movement provided additional evidence to support that mRNA exchange in these *in vitro* grafts was extensive and on a genome-wide scale.

Among the 172 transmitting genes whose mRNAs were found in both *in vitro* scions, three biological processes, responses to cadmium ion, metal ion and inorganic substance, were over-represented (Additional file 2: Dataset S5). In contrast, among the 80 transmitting genes whose mRNAs were transmitted to both *in vitro* rootstocks, five processes were over-represented. Four of the five processes were related to the biosynthesis of amine, amino acids, and nitrogen compounds. The other process was related to carbon utilization. Among the 28 transmitting genes whose mRNAs moved into both graft partners in the reciprocal grafts, amine and nitrogen compound biosynthesis were the two processes over-represented. In scions, genes for most biosynthetic pathways are expected to be very active. Therefore, these genes might be abundantly expressed and, as a result, transcripts from some of these genes were “spilled-over” and moved into the rootstocks. Likewise, in rootstocks, genes for acquiring ions and inorganic nutrients are expected to be active, thus explaining why transcripts from some of these genes moved into the scions.

We further analyzed the 107 genes whose mRNAs moved bi-directionally in the graft of *V. girdiana* (scion)/*V. palmata* (rootstock) (Fig. 2a). Nine over-represented biological processes were identified, including responses to light stimulus, radiation, and other forms of abiotic stresses and processes related to translation elongation and photosynthesis (Additional file 2: Dataset S5). A similar analysis on the 126 genes, whose mRNAs moved bi-directionally in the graft of *V. palmata* (scion)/*V. girdiana* (rootstock), revealed that only the phagocytosis process was over-represented. It appeared that the over-represented processes by the bi-directionally transmitting genes between the reciprocal grafts were very different.

There are different sets of unique genes moving into *V. girdiana* from *V. palmata*, depending on the graft combinations. When *V. girdiana* was used as the scion (1130 genes, Fig. 2a), many processes including signal transduction, regulation of response to stimulus, protein catabolic process, proteolysis and intracellular signaling cascade were over-represented (Additional file 2: Dataset S5). In contrast, when *V. girdiana* was used as the rootstock, over-represented processes were different and included intracellular transport, cellular component organization, mRNA metabolic process, and carbohydrate catabolic process. It appears that mRNA species being moved into a graft partner was affected by its role as a scion or rootstock in the graft combination. Similar observations were also obtained when mRNAs moving into *V. palmata* from *V. girdiana* were analyzed (Additional file 2: Dataset S5).

### mRNA movement in the field grafts

The field grafts, in which the wine grape scion of *V. vinifera* cultivar ‘Riesling’ was grafted onto the hybrid rootstock ‘C3309’, were grown under two soil conditions in field: untreated soil with a pH of 5.5 and treated soil with a pH of 6.5 (Table 1). The untreated soil with low pH is considered to be acidic for growing *V. vinifera* varieties [40]. A total of 987 transmitting genes were identified. Among them, 295 (about 30 %) transmitted transcripts in both soil conditions. While hundreds of scion mRNA species were found to move into the rootstock tissue under each soil condition (555 genes at the pH of 5.5 and 517 at the pH of 6.5), much fewer rootstock mRNA species (80 genes at the pH of 5.5 and 134 at the pH of 6.5) were detected in the sampled scion tissues (Fig. 2b). As what was observed in the *in vitro* grafts, some of the mobile mRNAs were transmitted bi-directionally to both scion and rootstock, with three in soil with the pH of 5.5 and five with the pH of 6.5. Among the mRNAs of the scion ‘Riesling’ moving into the rootstock ‘C3309’, on average about 45 % of them were shared between the grafts grown under the two soil conditions, demonstrating the reproducibility of our approach in detecting transmitting mRNAs. Interestingly, a similar percentage (45 %) of mobile mRNAs from the rootstock ‘C3309’ were also found in the scion ‘Riesling’ under both growing conditions (Fig. 2b).

GO term analysis of the 987 transmitting genes in the field grafts revealed that 69 processes were over-represented (Additional file 2: Dataset S6). Eleven of the processes were related to responses to certain forms of stimuli and stresses, such as water, temperature, chemicals and organic substances. It was interesting to note that these forms of stimuli, with the exception of water, were different from those observed in the *in vitro* grafts in which the transmitting genes were responsive to ions and hormone.

We examined biological significances of the mobile mRNAs detected in the grafts grown in two different pH soil conditions. First, GO term analysis was conducted on the 80 and 134 genes (Fig. 2b) transmitting their mRNAs from rootstocks to scions in the field grafts grown in the soil with a pH of 5.5 and 6.5, respectively. There were no apparently over-represented biological processes in the soil with the pH of 5.5, while several processes of cellular component assembly, protein polymerization, and molecular complex subunit organization were over-represented in the soil with the pH of 6.5.

We then analyzed the genes transmitting mRNAs to the rootstocks grown in the soil conditions with a pH of 5.5 (555 genes) and pH of 6.5 (517 genes) (Additional file 2: Dataset S6). In the soil with a pH of 5.5, a total of 40 processes were over-represented. Many of them were responsive to various forms of stresses, including water, heat, temperature, chemicals, hormone and others. Other over-represented processes included those involved in cellular, metabolic and biosynthetic activities. In contrast, in the soil with a pH of 6.5, 26 processes were over-represented and the majority of them were related to primary, cellular, protein, and lipid metabolic processes. We further examined the biological processes for those transmitting genes specific to a particular soil condition. In the soil with a pH of 5.5, 123 unique transmitting genes were identified. These genes were not found transmitting mRNAs in any other grafts in this study. Four processes were over-represented and all of them were related to some forms of responses to stresses, including light intensity, temperature, abscisic acid and other abiotic stimulus. In contrast, only 36 unique transmitting genes were identified in the soil with a pH of 6.5. Only the process of negative regulation of RNA metabolic process was over-represented. We also compared the expression level for the genes whose mRNAs were detected to transmit in only one soil condition. The majority of the genes had similar expression level in the source tissues under the two soil conditions (Additional file 1: Figure S4), indicating that selective transmissibility under different soil conditions were largely not due to differential gene expression in the source tissues. These results altogether suggested that the types of mRNAs involved in the movement between graft partners were very much affected by the environmental conditions under which the grafts were grown. In this case, genes responsive to various forms of stresses were presumably activated in the grafts grown in the soil with a pH of 5.5, compared to that in the soil with a pH of 6.5.

### mRNA movement in both *in vitro* and field grafts

There were 333 transmitting genes shared between the *in vitro* and field grafts investigated under this study. Among the 2083 rootstock genes whose mRNAs were found in the scions of *in vitro* grafts, 39 and 228 were overlapped with the transmitting genes of field rootstocks and scions, respectively (Fig. 2c and Additional file 2: Dataset S2). Similarly, among the 1313 scion genes whose mobile mRNAs were found in the rootstocks of *in vitro* grafts, 182 and 34 were overlapped with the transmitting genes of field scions and rootstocks, respectively. One gene, *GSVIVG01011146001* encoding a homeobox protein BEL1 homolog, was found to transmit mRNAs in both scions and rootstocks in both the *in vitro* and field grafts (Fig. 2c and Additional file 2: Dataset S2).

We were interested in examining whether or not some general biological processes were shared by the transmitting genes in both the *in vitro* and field grafts. There were 47 biological processes over-represented in the 333 shared transmitting genes (Additional file 2: Dataset S3). They included metabolic processes, cellular component assembly, biopolymer biosynthesis, macromolecular complex assembly, translation and regulation of many diverse processes. We then examined the 39 transmitting genes whose mRNAs moved from rootstocks to scions in both the *in vitro* and field grafts (Fig. 2c) and found that two processes, protein polymerization and translation, were over-represented (Additional file 2: Dataset S3). Among the 182 transmitting genes whose mRNAs moved from scions to rootstocks in both the *in vitro* and field grafts (Fig. 2c), macromolecule and cellular macromolecule metabolic processes were over-represented. Interestingly, the processes related to responses to various stresses were over-represented in both the *in vitro* and field transmitting genes, as described earlier, but such over-representation was not apparent when the *in vitro* and field shared transmitting genes were compared. This was likely due to the fact that the stress regimes were different between the *in vitro* and field grafts.

We further compared the transmitting genes identified in this study with those recently found in *Arabidopsis* grafts [33]. Orthologous genes between *Arabidopsis* and grape were identified using OrthoMCL [41]. We found that about 600 of the transmitting genes were overlapped between these two species. GO term analysis of these genes identified a large number of over-represented function terms related to many types of transmembrane transporter activities. In addition, we also identified several interesting over-represented biological processes including responses to certain forms of stresses and stimuli, hormone transport and signal transduction processes (Additional file 2: Dataset S7).

### Transmission rates of mobile mRNAs

**Fig. 3:**
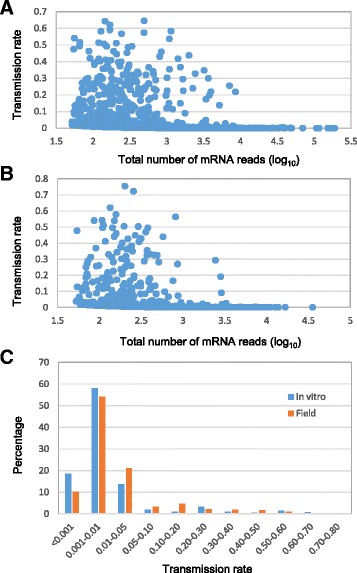
mRNA transmission rates and their distribution patterns. (**a**) Plot of transmission rates and the total numbers of mRNA reads (log_10_) detected for the 3115 *in vitro* graft transmitting genes. (**b**) Plot of transmission rates and the total numbers of mRNA reads (log_10_) detected for the 919 field graft transmitting genes. (**c**) Distribution of the transmission rates of mobile mRNAs from the transmitting genes identified from various rootstocks and scions in the *in vitro* (*n* = 3115) and field (*n* = 919) grafts. These genes had 50 or more RNA-Seq reads detected in the donor tissue and some of them may be represented by multiple data points if donor RNA-seq reads were detected in multiple receptor tissues

Transmission rates of mobile mRNAs could be influenced by genotypes, graft partners, growing conditions and other factors. We estimated the mRNA transmission rate of a mobile transcript by dividing the normalized number of donor RNA-Seq reads at a specific diagnostic SNP locus detected in the receptor tissue by the total normalized number of the donor RNA-Seq reads detected at that locus in both donor and receptor tissues. If mobile RNA-Seq reads were detected at multiple diagnostic SNP loci, the transmission rate of this transcript was estimated as the average of the transmission rates across all the individual SNP loci located in the gene (Fig. 3, Additional file 1: Figure S3). We recognize that our method for estimating transmission rates of mobile mRNAs has certain limits. For example, some genes may have different patterns in their temporal and spatial expression in the donor plants and the stability of mRNAs of these genes may differ in the receptor plants, therefore the amounts of mRNA in these different sample fractions are not necessarily comparable. Furthermore, our detection method are dependent on sequencing coverage and the presence of diagnostic SNPs between donor and receptor plants. Therefore, we will not be able to detect mobile mRNAs from those genes or coding regions of genes carrying no SNPs and/or with low coverage. Nevertheless, our estimates should provide a general pattern of this complex subject. To reduce potential bias due to small sample size, we estimated transmission rates for the graft transmitting genes with 50 or more RNA-Seq reads produced in the donor tissue. The mRNA transmission rates for these genes varied significantly, ranging from about 0.00001 to 0.6442 for the *in vitro* grafts (Fig. 3a) and from 0.00009 to 0.7554 for the field grafts (Fig. 3b). About 75 % and 65 % of the transmitting genes in the *in vitro* and field grafts, respectively, had mRNA transmission rates lower than 0.01 (Fig. 3c). In contrast, less than 2 % of the transmitting genes in both the *in vitro* and field grafts had the transmission rates higher than 0.50. It appeared that transmitting genes in the field grafts on average had higher transmission rates (0.0420) than that in the *in vitro* grafts (0.0238) (Table 1). This difference could be due to many factors including genotypes, age of grafted plants, tissue sampling, and growth environments (Additional file 1: Figure S1).

**Fig. 4:**
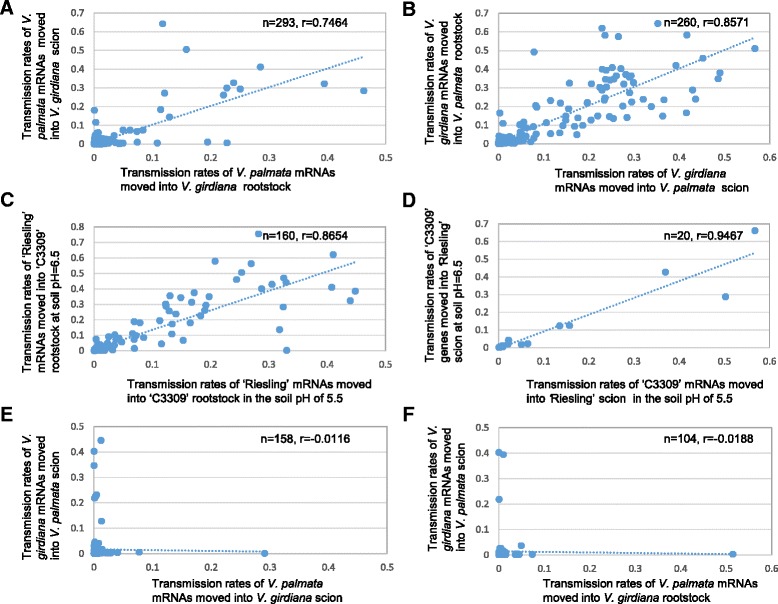
Plots of mRNA transmission rates of same genes in different graft tissues. Only genes with 50 or more RNA-Seq reads produced in the donor tissue were included. (**a**) Transmission rates of *V. palmata* mRNAs moved into *V. girdiana* rootstock vs. transmission rates of *V. palmata* mRNAs moved into *V. girdiana* scion. (**b**) Transmission rates of *V. girdiana* mRNAs moved into *V. palmata* scion vs. transmission rates of *V. girdiana* mRNAs moved into *V. palmata* rootstock. (**c**) Transmission rates of ‘Riesling’ mRNAs moved into ‘C3309’ rootstock in the soil with a pH of 5.5 vs. transmission rates of ‘Riesling’ mRNAs moved into ‘C3309’ rootstock in the soil with a pH of 6.5. (**d**) Transmission rates of ‘C3309’ mRNAs moved into *‘*Riesling’ scion in the soil with a pH of 5.5 vs. transmission rates of ‘C3309’ mRNAs moved into *‘*Riesling’ scion in the soil with a pH of 6.5. (**e**) Transmission rates of *V. palmata* mRNAs moved into *V. girdiana* scion vs. transmission rates of *V. girdiana* mRNAs moved into *V. palmata* scion. (**f**) Transmission rates of *V. palmata* mRNAs moved into *V. girdiana* rootstock vs. transmission rates of *V. girdiana* mRNAs moved into *V. palmata* scion

While there were exceptions, mRNA transmission rates of the same genes from the same genotype were generally correlated well, regardless whether the genotype was used as a rootstock or a scion (Fig. 4a and b). Among the 293 *V. palmata* genes examined with their mRNAs moved into both *V. girdiana* rootstocks and scions in the reciprocal grafts, the pairwise correlation coefficient of the mRNA transmission rates for these genes in the two graft tissues was 0.7464. A similar pairwise correlation coefficient (*r* = 0.8571) was found for the 260 *V. girdiana* genes with their mRNAs transmitted into both *V. palmata* rootstocks and scions. Such correlation relationships were also observed for field graft transmitting genes under different soil conditions (Fig. 4c and d). However, when the mRNA transmission rates of same genes from different genotypes were compared, no significant correlations (*r* < 0.02) were found (Fig. 4e and f).

### Potential mechanisms for long-distance mRNA movement

Transmission of mRNAs across graft junctions could be passive and/or selective. We observed evidences to support both modes of transmissions in this study. As described earlier, a large number of transmitting genes in both *in vitro* and field grafts had very low mRNA transmission rates (Fig. 3). Many of these genes had thousands of RNA-Seq reads in the donor tissue, but had only few of the donor mRNAs detected in the receptor tissue (Additional file 2: Dataset S2), suggesting that the mRNA movement of these genes was probably passive and likely a result of random movement processes. Another evidence to support the existence of a passive mechanism of mRNA movement was that highly expressed genes appeared to have higher chances of their transcripts transmitted and detected. We specifically examined 33 highly expressed genes (with RPKM value 1000 or more) in the *in vitro* grafts and found that 17 of them produced mobile mRNAs (Additional file 2: Dataset 2). The fact that most of these highly expressed genes generated mobile mRNAs strongly suggests the involvement of a mass flow mechanism in the mRNA movement.

While a mass flow or passive mechanism was apparently involved in the mRNA movement, convincing evidence was also found to support the presence of certain selective processes in facilitating transmissions of mRNAs across graft junctions. There were many transmitting genes whose mRNA transmission rates were relatively high and independent of their expression levels in both the *in vitro* and field grafts (Fig. 3a and b). This could not be simply explained by a random movement process. Instead, these transmitting genes were likely subject to certain selective processes for transmitting their mRNAs. When examining the 265 field-graft transmitting genes which had 50 or more RNA-Seq reads and transmission rates of 0.1 or higher, we observed that 15 biological processes, including phosphoinositide phosphorylation and metabolic process, were over-represented. Polyphosphoinositides are membrane lipids and play significant roles in osmotic stress signaling [42]. Over-representation of these stress-related processes might indicate that the related mRNA exchange was a response of the grafts to various field growth conditions, such as soil acidity. In addition, we compared the transmission rates of 160 genes whose mRNAs were found to be transmitted to the rootstock ‘C3309’ from the scion ‘Riesling’ in the field grafts grown under two different soil conditions (Fig. 4c). We found that although the transmission rates of the 160 genes in the grafts under the two soil conditions were highly correlated (*r* = 0.8654), about 10 % of the genes did show large differences in their transmission rates (5 times or more,). The cause for these apparent differences was not known, but some selective processes due to different field growing conditions were presumably involved. The selective processes became also apparent in the field grafts in which a significant number of mRNAs were transmitted only under a specific soil condition (Fig. 2b, Additional file 2; Datasets S2 and S6).

As shown earlier, much more mobile mRNAs were detected in the *in vitro* grafts than in the field grafts (Table 1). However, some graft transmitting genes were only identified in the field grafts, but not in the *in vitro* grafts even though these genes had comparable expression levels and diagnostic SNPs in both grafts. Examples include a putative transcription factor gene (*GSVIVG01023283001*), a putative E3 ubiquitin ligase gene (*GSVIVG01003757001*), a ring finger protein gene (*GSVIVG01026703001*), and a CBS domain protein gene (*GSVIVG01024516001*) (Additional file 2: Dataset S2). While we do not know the physiological significances of these genes, the observation of such genes being transmitted in the field grafts but not the *in vitro* grafts provided further evidence for the involvement of selective mechanisms in long distance mRNA movement in grapevine.

## Discussion

Examples of mRNA movement across graft junctions were previously demonstrated in several model plant species [15, 19, 21, 38]. Long distance movement of a few mRNA species has also been documented in apple, including *IAA14* and *GAI* [16, 17, 39]. Recently, extensive mRNA exchange was revealed between *Arabidopsis* and its parasitic plant *C. pentagona* through symplastic junctions [30, 31], between inter-generic grafts of *Arabidopsis* and tobacco [32], and between intra-specific (inter-ecotype) grafts of *Arabidopsis* through graft junctions [33]. However, these works were based on model and short-lived annual species and to what extent the conclusions from these studies can be applied to graft crops of economic significance is unknown. In this study, we advanced our knowledge in this area by extending the studies of mRNA exchange in model species to an important woody, fruit crop species of grapevines.

### Genome-wide exchanges of mRNAs between graft partners

A total of 3333 annotated grape genes were found to produce mobile mRNAs across graft junctions in this study. They accounted for about 12.7 % of the total protein coding genes (26,346) in grape. The extent of mRNA exchange between graft partners revealed in this study was extensive, at a similar scale as what was recently reported in *Arabidopsis* (about 6 %, 2006 out of 33,602 genes, produced mobile mRNAs) [33]. Because detection of mobile RNAs is contingent on the availability of SNPs differentiating graft partners, sequencing coverage, mRNA stability, tissue sampling and other technical and biological factors, it would not be possible to detect all the mobile mRNAs and, therefore, the proportion of the genes that were found to produce mobile mRNAs in this study is likely underestimated.

A significant portion of the transmitting genes showed very low mRNA transmission rates in this study (Fig. 3). Because only a small number of mobile mRNAs were present in the receptor tissue, their biological significances, if any, were difficult to assess. However, there were some genes which transmitted their mRNAs with relatively high rates in different grafts. These mobile mRNAs, while their biological significances were unknown, were likely transmitted through certain selective processes. Conceivably, the numbers and species of mRNAs which are responsive to selective translocation will be different under different growth conditions. Another interesting observation in this study was that the mRNA transmission rates of the same genes from the same genotype were generally correlated well, but not so evident between different genotypes. This suggests that the donor genotype likely plays a key role in determining how frequently mobile transcripts are transmitted in a graft.

The transmitting genes discovered in this study were involved in many different biological processes (Additional file 2: Datasets S3, S5 and S6). Many of these processes were over-represented in both the *in vitro* and field graft transmitted genes, covering various basic cellular, biosynthetic, catabolic, and metabolic activities. It was interesting to note that many processes related to responses to various forms of stresses and stimuli, such as water, temperature and chemicals, were over-represented, suggesting that mRNA movement in the grafted grapevines in this study were responsive to growth conditions and environmental stresses. Additional evidence to support this hypothesis is that the *in vitro* and field grafts which were grown under different stress regimes had unique, additional stress-responsive genes involved. In the field grafts, mobile mRNAs from genes which were responsive to the stimulus of abscisic acid, carbohydrate, chitin, and organic substance were uniquely over-represented. In contrast, in the *in vitro* grafts, mRNAs from the genes responsive to cadmium ion, hormone, inorganic substance, metal ion, and salt stress were over-represented. In addition to this stress-responsive theme, we also found that many transcription factors and hormone-related genes participated in long-distance mRNA transmission, which presumably provide additional levels of regulations of many plant growth and development processes in the grafted plants.

We discovered that there were about 600 transmitting genes shared between the grapevines in this study and the *Arabidopsis* previously reported [33]. While these shared genes had diverse functions and were involved in many different biological processes, some of them were related to hormone transport, signal transduction and responses to certain forms of stresses and stimuli. Whether or not some of these genes are representative of the core common genes involved in producing and transmitting mRNAs in grafted plants is yet to be confirmed.

### Impact of graft combinations, genotypes, and growth conditions on mRNA exchange

Impact of scion/rootstock combinations on macromolecular translocation has been reported before. The study on the graft transmission of phloem proteins in interspecific and intergeneric heterografts in the *Cucurbitaceae* family suggested that the direction of phloem protein translocation depended on the scion/rootstock combination [43]. Similarly, the *mouse ear* tomato mutant can induce leaf phenotypic changes in wild-type grafting partner only when the mutant was used as the rootstock [22]. On the other hand, *in vitro* reciprocal grafts between wild type and transgenic potato plants overexpressing the *POTH1* gene demonstrated that the transgenic *POTH1* only moved toward the rootstock [14]. Both directional and bi-directional exchanges of mRNAs between rootstocks and scions took place in grafted *Arabidopsis* [33]. We also observed such directional and bi-directional exchanges of mRNAs in the grafted grapevines in this study (Fig. 2), providing first support evidence from a woody species.

Overall, the number of mobile RNAs found in the field grafts was much smaller than that in the *in vitro* grafts. In addition, we observed that more rootstock mRNAs moved into the scion tissues in the *in vitro* reciprocal grafts. However, a reversed case was found in the field grafts. These differences could be attributed to different graft genotypes, different growth conditions (*in vitro* vs. field), different ages of graft material (4 weeks *in vitro* vs. 11 years in field), and different proximities of the scion and rootstock tissues to the graft junctions (few centimeters *in vitro* vs. several meters in field) (Additional file 1: Figure S1). Moreover, the *in vitro* grafts were grown on growth medium containing sucrose and other nutrients, thus the source-sink gradient for the *in vitro* grafts was not as apparent and effective as that in the field grafts. Furthermore, in the mature field grafts, mobile mRNAs from rootstocks would have to travel over a long distance to reach young scion shoots and therefore many of the mobile mRNAs from rootstocks might not reach that far before being degraded. Indeed, investigation of the distribution of a particular tomato host gene with high level of mobility along the stem of the parasitic plant (*C. pentagona*) revealed that the host gene transcript level decreased significantly from the basal section to the apical tip [30]. A similar gradient for RNA movement was also reported in *Arabidopsis* grafts [33]. These findings suggest that most mRNA species in the phloem stream might not be very stable or did not diffuse or migrate very far from the site where the message was generated, which offers a plausible explanation of why so few mobile RNAs were detected in the scion tissue of the field grafts in this study. Comparisons of the abundance, movement directions and patterns of mobile mRNAs in the *in vitro* and field grafts revealed an important fact that while many hundreds, perhaps even thousands, of genes could transmit their mRNAs between graft partners, only a small number of them might reach certain tissues to become biologically relevant. Such comparisons also reinforced that research results of mRNA exchange from model species and certain experimental material, such as the *in vitro* grafts in this study, were invaluable, but special cautions are needed to interpret the results, especially when extending the conclusions beyond the system studied.

Genotypes, scion/rootstock combinations, and growth conditions not only affected the scale or extent of the mRNA exchange, but also had significant impact on the species of mRNAs transmitted. We revealed that many biological processes conferred by the mobile mRNAs were shared by different genotypes, graft partners, and grafts grown in different conditions, but at the same time, there were many processes uniquely over-represented under certain biological and environmental conditions. The genetic and physiological bases for these graft-, genotype- and environment-dependent mRNA movements are yet to be elucidated. Future studies in this area are certainly of great interest not only to the understanding of the molecular and genetic mechanisms regulating the process of mRNA movement in grafted plants, but also to the development and selection of superior grafts for practical agricultural uses.

### mRNA movement mechanisms

While many mRNAs were detected in phloem saps in plants [8, 9, 11, 12, 38, 39], few were found with known necessity of long distance trafficking to carry out their functions. A closer examination of the macromolecules detected in phloem sap showed that many of these molecules are quite abundant in plants in general [44–46]. Many components for protein translation and protein degradation were detected in phloem stream but may have no necessary function there and were ‘leaked’ or diffused passively into the phloem stream simply due to their abundant quantity in plants [1]. All these work suggested that ‘spill over’ was likely a cause for the presence of a large number of macromolecules, including mRNAs, in the plant phloem system. The detection of host non-phloem mobile transcripts in the parasitic plant tissues of *C. pentagona* also provided supporting evidence of such possible ‘spill over’ of abundant transcripts from cells to cells [30, 31]. The fact that more than 10 % of the graft transmitting genes showed very low transmission rates in this study (less than 0.001, Fig. 3c) also suggests the existence of a genome-wide, non-selective mass flow mechanism involved in the mRNA movement across graft junctions of grapevines (Fig. 3). For example, many genes coding for ribosome components were found to transmit their mRNAs across graft junctions at low transmission rates and they were over-represented in both field and *in vitro* graft transmitted genes (Additional file 2: Datasets S3, S5 and S6). This structural constituent of ribosome is a common component observed in almost all studies of phloem mRNA populations [8, 9, 11, 39]. The presence of such mRNAs in phloem sap samples could be explained by the passive mass flow into the phloem stream, since many of those transcripts were ubiquitously expressed in all plant parts and there would be no biological necessity for such messages to be selectively translocated across the graft junction into a grafting partner.

Active long-distance mRNA trafficking has been reported for *StBEL5* in potato and *GAI* in *Arabidopsis* [15, 20, 21]. Selective movement of mRNAs across graft junctions was recently demonstrated in the grafted *Arabidopsis* [33]. In this study, we observed that some genes had their mobile RNAs detected in the field grafts, but not in the *in vitro* grafts, even though they had diagnostic SNPs and were expressed at comparable levels as that in the field grafts. Why the mRNAs of these genes were transmitted across graft junctions in the field grafts, not in the *in vitro* grafts, is unknown, but some types of selective processes, including those dependent on genotypes, growth conditions and age of grafts, might have been involved. We also observed that some genes produced mobile mRNAs transmitted only to one graft partner, but not to the other, suggesting that some selective processes might be involved in promoting or inhibiting the movement of these mRNAs towards a particular graft partner or direction. Additional evidence for supporting the existence of selective process in mobile mRNA movement in this study came from the fact that some genes were expressed at relative low levels, but their mRNAs were transmitted at the rates higher than expected by random transmission (Fig. 3). These genes might contain some intrinsic elements facilitating their long distance trafficking as reported by others [21, 25].

## Conclusions

Although grafting has been practiced in many fruit species for centuries, how genetically the graft partners interact with each other to produce vigorous grafted plants is largely unknown. Conceivably, the large genomic-scale exchanges of mRNAs between scions and rootstocks, as found in this study, could be the key genetic basis of superior performance of grafted plants. While the relative transmission rates of mRNAs were generally low, 3333 grape genes involved in such large-scale exchanges of genetic information between scions and rootstocks underscored the underlying importance of the phenomenon. As revealed in this study, such exchanges could be affected by genetic, genotypic, environmental factors and possibly controlled by different mRNA movement mechanisms. While many biological processes and mechanisms involved in such mRNA movement are yet to be elucidated, one obvious benefit from such exchange of mRNAs between two genetically distinct graft partners would increase diversity of the mRNA pool accessible to both scion and rootstock in a graft. Such diverse pool of mRNAs in turn can make the grafted plants more productive and adaptive to various biotic and abiotic conditions through complementation and synergistic interactions of the two genetic systems from scions and rootstocks.

## Methods

### *In vitro* material

To mimic commercially grafted grapevines, all grafts evaluated in this study were made between different *Vitis* species. Two wild *Vitis* species, *V. girdiana* and *V. palmata*, were cultured *in vitro* and maintained using a protocol previously reported [47]. *In vitro* plants of 8-week old were harvested from the culture. All visible leaves and roots were removed and the remaining stems and shoots were used as either scions or rootstocks in grafting. Briefly, axillary shoots with approximately 2-cm long stems containing 1–2 lateral buds were used as scions. Older stems of approximately 3-cm long were used for rootstocks. The scion and the rootstock were then cleft-grafted together and the grafting union was gently wrapped with wet stripes of sterile Kimwipes. The grafts were placed vertically on micropropagation medium in a Magenta box, with the grafting union being at least 1 cm above the medium. The grafts were cultured in Magenta boxes in a tissue culture room until tissues were collected for RNA extraction. Grafted plants were taken out of Magenta boxes and the graft junction of a graft plant was first excised out with approximately 0.5-cm long stem on each side of the junction. The graft junction was not included for RNA extraction (Additional file 1: Figure S1). Tissues were harvested at four weeks post grafting. The freshly collected tissues were flash frozen in liquid nitrogen and then stored in−80 °C freezer.

### Field grafting material

The field graft material was sampled from an established rootstock trial at the Fredonia Vineyard Laboratory of the Cornell University in Fredonia, New York [40]. The rootstock trial contained four replicates of grafts involving four scions and four rootstocks grown in two different soil conditions (untreated soil with pH of 5.0–5.5 and limestone-treated soil with pH of 6.0–6.5; for brevity, pH of 5.5 and pH of 6.5 used thereafter). Scion canes were bench grafted to rootstocks. Grafted grapevines were grown in a nursery for the first year. One-year-old dormant grafted vines were then planted in the field in 2003. These trial vines were maintained following general viticultural management. A subset of the rootstock trial materials, *V. vinifera* var. ‘Riesling’ scion grafted to the ‘C3309’ rootstock, was sampled for the present study. The scion ‘Riesling’ is a well-recognized wine grape cultivar of *V. vinifera* which is the most widely cultivated grape species. The rootstock ‘C3309’ is an inter-specific *Vitis* hybrid derived from a cross between two closely related species *V. riparia* and *V. rupestris* (http://iv.ucdavis.edu/Viticultural_Information/?uid=172&ds=351). Young shoots (including shoot tips and developing leaves) and tertiary roots were collected from field grafts in August of 2013 (Table 1 and Additional file 1: Figure S1). The collected tissues were immediately immersed into RNA Later solution (Qiagen) in 15 ml falcon tubes stored on wet ice and later stored in a−20 °C freezer.

### Preparation of strand-specific RNA sequencing libraries

To avoid potential contamination among different genotypes, we processed samples of individual genotypes, one at a time, for tissue grinding, RNA extraction, mRNA purification and mRNA library construction. Total RNAs were extracted using Spectrum Plant Total RNA Kit (Sigma-Aldrich). mRNAs were purified using Dynabeads Oligo d(T)_25_ (Life Technologies). Purified mRNAs were quantified using Quant-iT RNA assay kit with the Qubit fluorometer (Life Technologies). About 40 ng mRNAs were used for preparation of the strand-specific mRNA library following previously published protocols [48, 49]. Ten PCR cycles were used in the final amplification step. RNA-Seq libraries were sequenced using the Illumina HiSeq system at the Biotechnology Resource Center of Cornell University (Ithaca, NY, USA). To avoid potential contamination, each library was sequenced on a separate lane.

### Preparation of genomic libraries

Genomic DNA was extracted from young leaf tissues for each graft genotype using DNeasy Plant kit (Qiagen). About 100 ng genomic DNA was fragmented to 200–700 bp inserts with DNA fragmentase (NEB). Steps of end-repair, d(A)-tailing, adaptor ligation were the same as the ones for preparation of mRNA libraries. Eight PCR cycles were used for final library amplification. The genomic libraries were sequenced with the Illumina HiSeq system using the paired-end 100 bp or 151 bp mode. Each genotype has at least 60 million reads with genome coverage of 15× or more (Additional file 2: Dataset S1).

### Detection of mobile mRNAs transmitted between scion and rootstock of grafted plants

Genome sequencing data of *V. girdiana*, *V. palmata*, ‘C3309’ and ‘Riesling’ were used to compile the SNP loci between scion and rootstock. The genome reads were mapped to the *V. vinifera* reference genome (12X) [50] using BWA allowing up to four edit distances for 100-bp reads and six for 151-bp reads [51]. Only uniquely mapped reads (reads with one single best hit) were kept. Potential PCR duplicates were removed based on the mapping results. Following alignment, the coverage of each genomic position by base A, G, C and T was calculated based on the mpileup file generated by SAMtools [52]. Only loci which were homozygous and showed different genotypes between the scion and the rootstock of a grafted plant (diagnostic SNPs) were used for downstream transmitting locus identification. For each homozygous locus, we required at least seven reads supporting the dominant allele and the frequency of the dominant allele being greater than 90 %.

RNA-Seq reads from individual rootstocks and scions of various grafts were separately aligned to the *V. vinifera* reference genome and the read coverage of each genomic position by base A, G, C and T was similarly determined using the same method as described above. The transmission ability of a transcript was determined by comparison of corresponding genomic and RNA-Seq reads of rootstocks and scions. A transcript is defined as mobile if its corresponding RNA-Seq reads from the donor were detected in the receptor’s RNA-Seq library. These reads should be perfectly aligned to the donor genome, and the transcripts should meet at least one of the following criteria: (1) having at least one read carrying two or more diagnostic SNP loci (Fig. 1a); (2) having at least two unique reads covering one diagnostic SNP locus (Fig. 1b); or (3) having two or more unique reads carrying different diagnostic SNP loci (Fig. 1c). A gene which produces mobile transcripts between scion and rootstock is described thereafter as a graft transmitting gene.

### Estimation of transmission rates

We used a window-based approach to estimate the transmission rate of a mobile transcript between scion and rootstock. For each transmitted SNP locus, a window centered at the locus was generated. The window was extended to the left and right by a size of read length, respectively. To estimate the transmission rate of an mRNA transcript from donor to receptor, we counted the numbers of the donor and receptor RNA-Seq reads, respectively, which perfectly matched the donor genome within the transmission window (Additional file 1: Figure S3), and the raw counts were then normalized to RPM (read per million). The transmission rate for a specific mobile transcript was estimated by dividing the normalized number of donor RNA-Seq reads at a specific SNP locus detected in the receptor tissue by the total normalized number of the donor RNA-Seq reads detected at that locus in both donor and receptor tissues. If supporting RNA-Seq reads were detected at multiple diagnostic SNP loci, the transmission rate of this transcript was estimated as the mean of the transmission rates across all the individual SNP loci located in the gene.

### RNA-Seq gene expression analysis

RNA-Seq reads were first aligned to ribosomal RNA sequence database [53] using Bowtie allowing two mismatches [54] and those aligned were discarded. The resulting filtered reads were aligned to the *V. vinifera* reference genome using TopHat [55]. Following alignments, raw counts for each grape gene were derived and then normalized to reads per kilobase of exon model per million mapped reads (RPKM).

### Availability of supporting data

All relevant supporting data can be found within the additional files accompanying to this article. The raw genome sequencing and RNA-Seq reads have been deposited in NCBI SRA under the accession numbers SRP058158 and SRP058157, respectively.

## Additional files

Additional file 1: Figure S1. Illustration of scion and rootstock tissues used in this study. **Figure S2.** Different types of RNA-Seq reads and their contributions to mobile mRNA detection. **Figure S3.** A window-based approach to estimate transmission rates. **Figure S4.** Scatter plot of expression levels in source tissues at two different soil conditions (pH of 5.5 and 6.5) of genes whose mRNAs were detected to transmit in only one of the two soil conditions. (PDF 653 kb) Additional file 2: Dataset S1. Summary of RNA-Seq and genome resequencing. **Dataset S2.** Summary of RNA-Seq reads detected in the donors (source) and receptors of various grafts. **Dataset S3.** Over-represented GO processes of the genes producing mobile mRNAs detected in different growth conditions, genotypes and tissue types. **Dataset S4.** Mobile mRNAs detected for the genes encoding transcription factors and hormone-related genes in this study. **Dataset S5.** Summary of over-represented GO terms in various *in vitro* grafts. **Dataset S6.** Summary of over-represented GO terms in various field grafts. **Dataset S7.** Over-represented GO terms of graft transmitting genes shared by grapevine and Arabidopsis. (XLSX 896 kb)

## Abbreviations

mRNA: Messenger ribonucleic acid
RNA-seq: RNA sequencing
POTH1: Potato homeobox 1
GO: Gene ontology
SNP: Single-nucleotide polymorphism
RPKM: Reads per kilobase of exon model per million mapped reads

## References

[1] Haroldsen VM, Szczerba MW, Aktas H, Lopez-Baltazar J, Odias MJ, Chi-Ham CL (2012). Mobility of transgenic nucleic acids and proteins within grafted rootstocks for agricultural improvement. Front Plant Sci.

[2] Mudge K, Janick J, Scofield S, Goldschmidt EE (2009). A history of grafting. Hortic Rev.

[3] Gale G, Pinder MSR (2002). Saving the vine from *Phylloxea*: a never-ending battle. Wine: A Scientific Exploration.

[4] Lucas WJ, Yoo BC, Kragler F (2001). RNA as a long-distance information macromolecule in plants. Nat Rev Mol Cell Bio.

[5] Turnbull CGN, Lopez-Cobollo RM (2013). Heavy traffic in the fast lane: long-distance signalling by macromolecules. New Phytol.

[6] Kollmann R, Dorr I, Kleinig H (1970). Protein filaments - structural components of phloem exudate.1. observations with Cucurbita and Nicotiana. Planta.

[7] Ziegler H, Zimmermann MH, Milburn JA (1975). Nature of transported substances in the phloem. Encyclopedia of Plant Physiology.

[8] Deeken R, Ache P, Kajahn I, Klinkenberg J, Bringmann G, Hedrich R (2008). Identification of Arabidopsis thaliana phloem RNAs provides a search criterion for phloem-based transcripts hidden in complex datasets of microarray experiments. Plant J.

[9] Doering-Saad C, Newbury HJ, Couldridge CE, Bale JS, Pritchard J (2006). A phloem-enriched cDNA library from Ricinus: insights into phloem function. J Exp Bot.

[10] Gaupels F, Buhtz A, Knauer T, Deshmukh S, Waller F, van Bel AJE (2008). Adaptation of aphid stylectomy for analyses of proteins and mRNAs in barley phloem sap. J Exp Bot.

[11] Omid A, Keilin T, Glass A, Leshkowitz D, Wolf S (2007). Characterization of phloem-sap transcription profile in melon plants. J Exp Bot.

[12] Sasaki T, Chino M, Hayashi H, Fujiwara T (1998). Detection of several mRNA species in rice phloem sap. Plant Cell Physiol.

[13] Notaguchi M, Wolf S, Lucas WJ (2012). Phloem-mobile Aux/IAA transcripts target to the root tip and modify root architecture. J Integr Plant Biol.

[14] Mahajan A, Bhogale S, Kang IH, Hannapel DJ, Banerjee AK (2012). The mRNA of a Knotted1-like transcription factor of potato is phloem mobile. Plant Mol Biol.

[15] Huang NC, Yu TS (2009). The sequences of Arabidopsis GA-INSENSITIVE RNA constitute the motifs that are necessary and sufficient for RNA long-distance trafficking. Plant J.

[16] Xu HY, Zhang WN, Li MF, Harada T, Han ZH, Li TZ (2010). Gibberellic acid insensitive mRNA transport in both directions between stock and scion in Malus. Tree Genet. Genomes.

[17] Xu HY, Iwashiro R, Li TZ, Harada T (2013). Long-distance transport of Gibberellic Acid Insensitive mRNA in Nicotiana benthamiana. Bmc Plant Biol.

[18] Kudo H, Harada T (2007). A graft-transmissible RNA from tomato rootstock changes leaf morphology of potato scion. Hortscience.

[19] Haywood V, Yu TS, Huang NC, Lucas WJ (2005). Phloem long-distance trafficking of Gibberellic acid-insensitive RNA regulates leaf development. Plant J.

[20] Hannapel DJ (2010). A model system of development regulated by the long-distance transport of mRNA. J Integr Plant Biol.

[21] Banerjee AK, Chatterjee M, Yu YY, Suh SG, Miller WA, Hannapel DJ (2006). Dynamics of a mobile RNA of potato involved in a long-distance signaling pathway. Plant Cell.

[22] Kim M, Canio W, Kessler S, Sinha N (2001). Developmental changes due to long-distance movement of a homeobox fusion transcript in tomato. Science.

[23] Kehr J (2009). Long-distance transport of macromolecules through the phloem. F1000 Biol Rep.

[24] Spiegelman Z, Golan G, Wolf S (2013). Don’t kill the messenger: long-distance trafficking of mRNA molecules. Plant Sci.

[25] Li CY, Zhang K, Zeng XW, Jackson S, Zhou Y, Hong YG (2009). A cis element within Flowering Locus T mRNA determines its mobility and facilitates trafficking of heterologous viral RNA. J Virol.

[26] Shin HI, Cho NJ, Cho TJ (2008). Role of 5 ’-UTR hairpins of the Turnip yellow mosaic virus RNA in replication and systemic movement. BMB Rep.

[27] Takeda R, Petrov AI, Leontis NB, Ding BA (2011). A three-dimensional RNA motif in potato spindle tuber viroid mediates trafficking from palisade mesophyll to spongy mesophyll in Nicotiana benthamiana. Plant Cell.

[28] Ham BK, Brandom JL, Xoconostle-Cazares B, Ringgold V, Lough TJ, Lucas WJ (2009). A polypyrimidine tract binding protein, pumpkin RBP50, forms the basis of a phloem-mobile ribonucleoprotein complex. Plant Cell.

[29] Xoconostle-Cazares B, Yu X, Ruiz-Medrano R, Wang HL, Monzer J, Yoo BC (1999). Plant paralog to viral movement protein that potentiates transport of mRNA into the phloem. Science.

[30] LeBlanc M, Kim G, Patel B, Stromberg V, Westwood J (2013). Quantification of tomato and Arabidopsis mobile RNAs trafficking into the parasitic plant Cuscuta pentagona. New Phytol.

[31] Kim G, LeBlanc ML, Wafula EK, dePamphilis CW, Westwood JH (2014). Genomic-scale exchange of mRNA between a parasitic plant and its hosts. Science.

[32] Notaguchi M, Higashiyama T, Suzuki T (2015). Identification of mRNAs that move over long distances using an RNA-Seq analysis of Arabidopsis/Nicotiana benthamiana heterografts. Plant Cell Physiol.

[33] Thieme CJ, Rojas-Triana M, Stecyk E, Schudoma C, Zhang W, Yang L, et al. Endogenous Arabidopsis messenger RNAs transported to distant tissues. Nature Plants 2015, 1:doi:10.1038/nplants.2015.1025. 10.1038/nplants.2015.25 27247031

[34] Joung JG, Corbett AM, Fellman SM, Tieman DM, Klee HJ, Giovannoni JJ (2009). Plant MetGenMAP: an integrative analysis system for plant systems biology. Plant Physiol.

[35] Bai X, Rivera-Vega L, Mamidala P, Bonello P, Herms DA, Mittapalli O (2011). Transcriptomic signatures of ash (Fraxinus spp.) phloem. Plos One.

[36] Guo S, Zhang J, Sun H, Salse J, Lucas WJ, Zhang H (2013). The draft genome of watermelon (Citrullus lanatus) and resequencing of 20 diverse accessions. Nat Genet.

[37] Ruiz-Medrano R, Xoconostle-Cazares B, Lucas WJ (1999). Phloem long-distance transport of CmNACP mRNA: implications for supracellular regulation in plants. Development.

[38] Yang HW, Yu TS (2010). Arabidopsis floral regulators FVE and AGL24 are phloem-mobile RNAs. Bot Stud.

[39] Kanehira A, Yamada K, Iwaya T, Tsuwamoto R, Kasai A, Nakazono M (2010). Apple phloem cells contain some mRNAs transported over long distances. Tree Genet Genomes.

[40] Bates TR, Walter-Peterson HC, Reisch BI, Dunst RM (2003). Improving wine grapes production in acid soils with rootstocks and soil management. Hortisci.

[41] Li L, Stoeckert CJ, Roos DS (2003). OrthoMCL: identification of ortholog groups for eukaryotic genomes. Genome Res.

[42] Munnik T, Vermeer JE (2010). Osmotic stress-induced phosphoinositide and inositol phosphate signalling in plants. Plant Cell Environ.

[43] Golecki B, Schulz A, Carstens-Behrens U, Kollmann R (1998). Evidence for graft transmission of structural phloem proteins or their precursors in heterografts of Cucurbitaceae. Planta.

[44] Lin MK, Lee YJ, Lough TJ, Phinney BS, Lucas WJ (2009). Analysis of the pumpkin phloem proteome provides insights into Angiosperm sieve tube function. Mol Cell Proteomics.

[45] Zhang S, Sun L, Kragler F (2009). The phloem-delivered RNA pool contains small noncoding RNAs and interferes with translation. Plant Physiol.

[46] Dinant S, Lemoine R (2010). The phloem pathway: new issues and old debates. Cr Biol.

[47] Jittayasothorn Y, Yang Y, Chen S, Wang X, Zhong GY (2011). Influences of Agrobacterium rhizogenes strains, plant genotypes, and tissue types on the induction of transgenic hairy roots in Vitis species. Vitis.

[48] Wang L, Si YQ, Dedow LK, Shao Y, Liu P, Brutnell TP. A Low-cost library construction protocol and data analysis pipeline for Illumina-based strand-specific multiplex RNA-Seq. Plos One 2011, 6(10):doi:10.1371/journal.pone.0026426. 10.1371/journal.pone.0026426 PMC3198403 22039485

[49] Zhong S, Joung JG, Zheng Y, Chen YR, Liu B, Shao Y (2011). High-throughput illumina strand-specific RNA sequencing library preparation. Cold Spring Harb Protoc.

[50] Jaillon O, Aury JM, Noel B, Policriti A, Clepet C, Casagrande A (2007). The grapevine genome sequence suggests ancestral hexaploidization in major angiosperm phyla. Nature.

[51] Li H, Durbin R (2009). Fast and accurate short read alignment with Burrows-Wheeler transform. Bioinformatics.

[52] Li H, Handsaker B, Wysoker A, Fennell T, Ruan J, Homer N (2009). The Sequence Alignment/Map format and SAMtools. Bioinformatics.

[53] Quast C, Pruesse E, Yilmaz P, Gerken J, Schweer T, Yarza P (2013). The SILVA ribosomal RNA gene database project: improved data processing and web-based tools. Nucleic Acids Res.

[54] Langmead B, Trapnell C, Pop M, Salzberg SL (2009). Ultrafast and memory-efficient alignment of short DNA sequences to the human genome. Genome Biol.

[55] Trapnell C, Pachter L, Salzberg SL (2009). TopHat: discovering splice junctions with RNA-Seq. Bioinformatics.

