# Supplementary material for: Messenger RNA exchange between scions and rootstocks in grafted grapevines
Source: BMC Plant Biol. 2015 Oct 19;15:251. doi: 10.1186/s12870-015-0626-y (PMC4612405; doi:10.1186/s12870-015-0626-y)
Supplement: Additional file 1: Figure S1. — Illustration of scion and rootstock tissues used in this study. Figure S2. Different types of RNA-Seq reads and their contributions to mobile mRNA detection. Figure S3. A window-based approach to estimate transmission rates. Figure S4. Scatter plot of expression levels in source tissues at two different soil conditions (pH of 5.5 and 6.5) of genes whose mRNAs were detected to transmit in only one of the two soil conditions. (PDF 653 kb) [file 12870_2015_626_MOESM1_ESM.pdf]

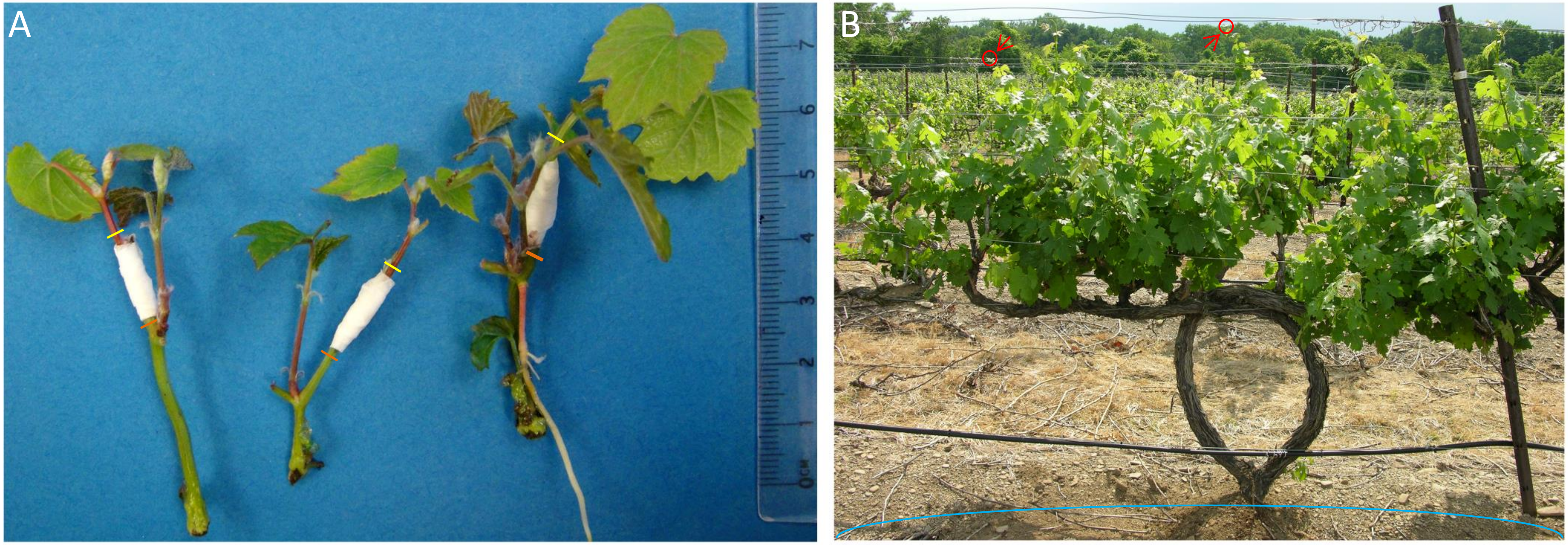

Figure S1. **Figure S1**. Illustration of scion and rootstock tissues used in this study. **A:** *in vitro* grafted grapevines. Grafted plants were grown in Magenta boxes and were sampled 4 weeks after grafting. The graft junction, which was wrapped with sterilized kimwipes, were excised out and not included in the study. Tissues collected above the yellow lines and below the orange lines were used as scion and rootstock material, respectively. **B:** Field grafted grapevines. The young shoots and leaves (red circles) were collected as scion tissue, and small tertiary roots, dug out from the soil (area marked with the blue arc), as rootstock tissue.

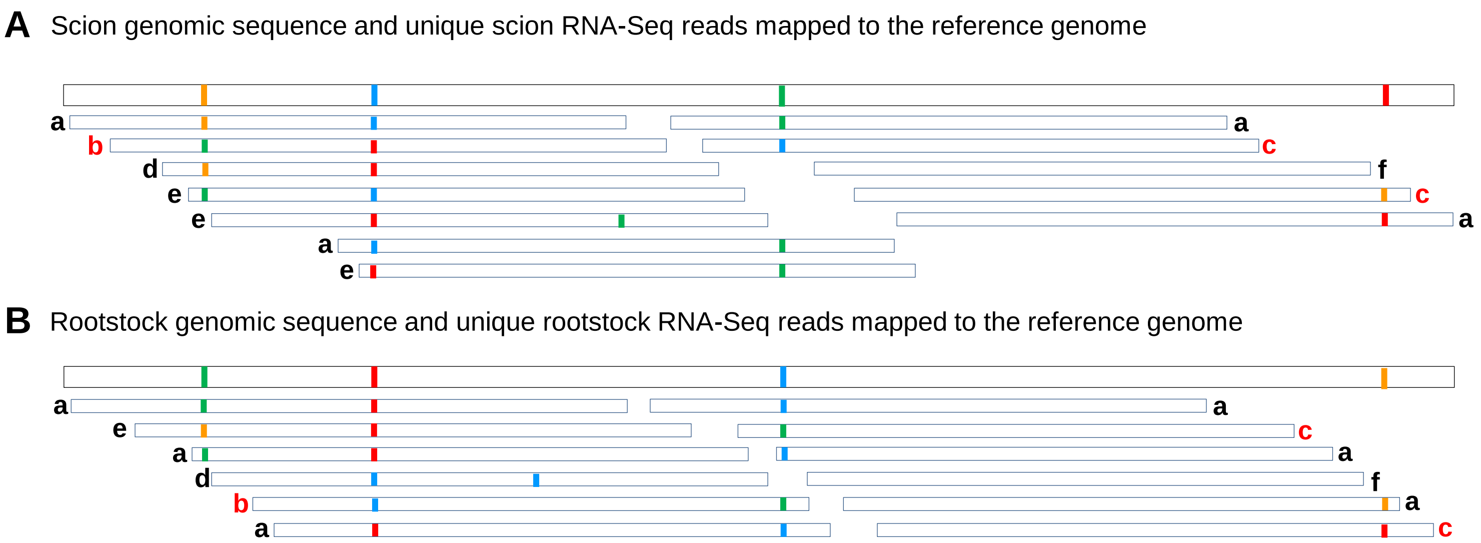

Figure S2. Different types of RNA-Seq reads and their contributions to mobile mRNA detection**. A**: unique RNA-Seq reads from scion RNA-Seq library mapped to the reference genome. The long bar on the top represents the corresponding scion genomic sequence derived from the scion genomic library. **B**: unique RNA-Seq reads from rootstock RNA-Seq library mapped to the reference genome. The long bar on the top represents the corresponding rootstock genomic sequence derived from the rootstock genomic library. SNPs between the scion and rootstock genomic sequences were marked with colored bars, orange for “G”, green for “A”, blue for “C” and red for “T”. In a receptor’s RNA-Seq library, most reads, as expected, carry the same SNPs as the receptor genotype (**a**-type); Other types of reads include: **b**-type carrying flanking donor-specific SNPs, **c**-type carrying only one donor-specific SNP, **d**-type carrying additional SNPs which was not present in donor genomic sequence, **e**-type carrying only one but not all donor-specific SNPs, and **f**-type which falls into the regions without diagnostic SNPs. mRNAs with **b**-type and **c**-type reads detected were be classified as putative graft mobile mRNAs . **d**-type and **e**-type reads will be eliminated in the pipeline since the reads do not match perfectly to the rootstock (donor) genomic sequence. They are likely due to sequencing or alignment error, RNA-editing or some unknown genetic factors. **f**-type reads fall into regions without SNPs and won’t contribute to the identification of mobile mRNAs .

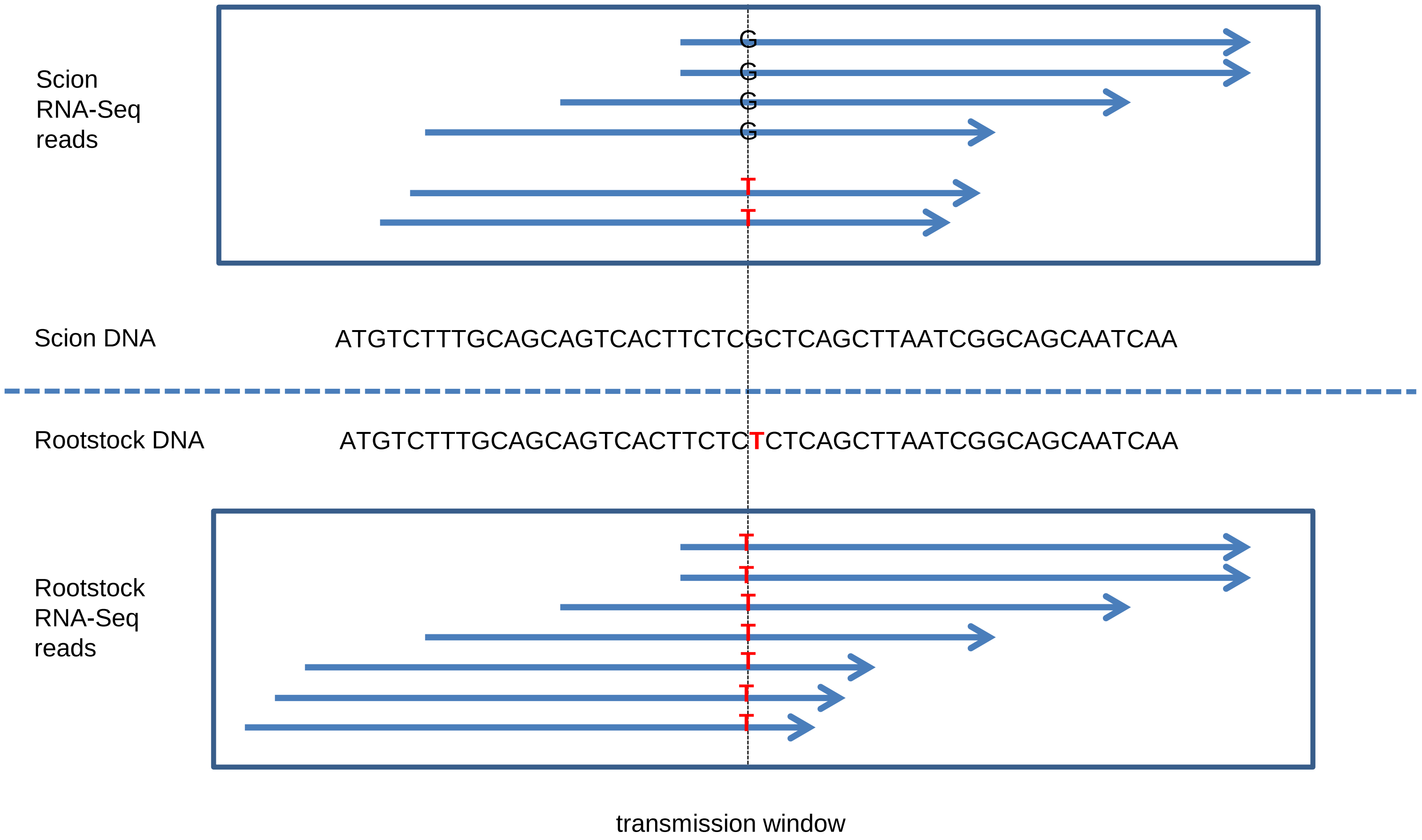

Figure S3. A window-based approach to estimate transmission rates. The vertical dashed line indicates a potential transmitting locus. The window was centered at the locus and then extended to the left and the right by a size of read length, respectively. In this example, among the 6 reads from the scion RNA-Seq library that were mapped within the window, two were from the rootstock while the other four originated from the scion. The transmission rate for this specific locus was calculated as the rootstock-specific reads in the receptor tissue (2) normalized to RPKM divided by the total rootstock-specific reads (7 reads from donor tissue normalized to RPKM plus 2 transmitted reads from the receptor tissue normalized to RPKM).

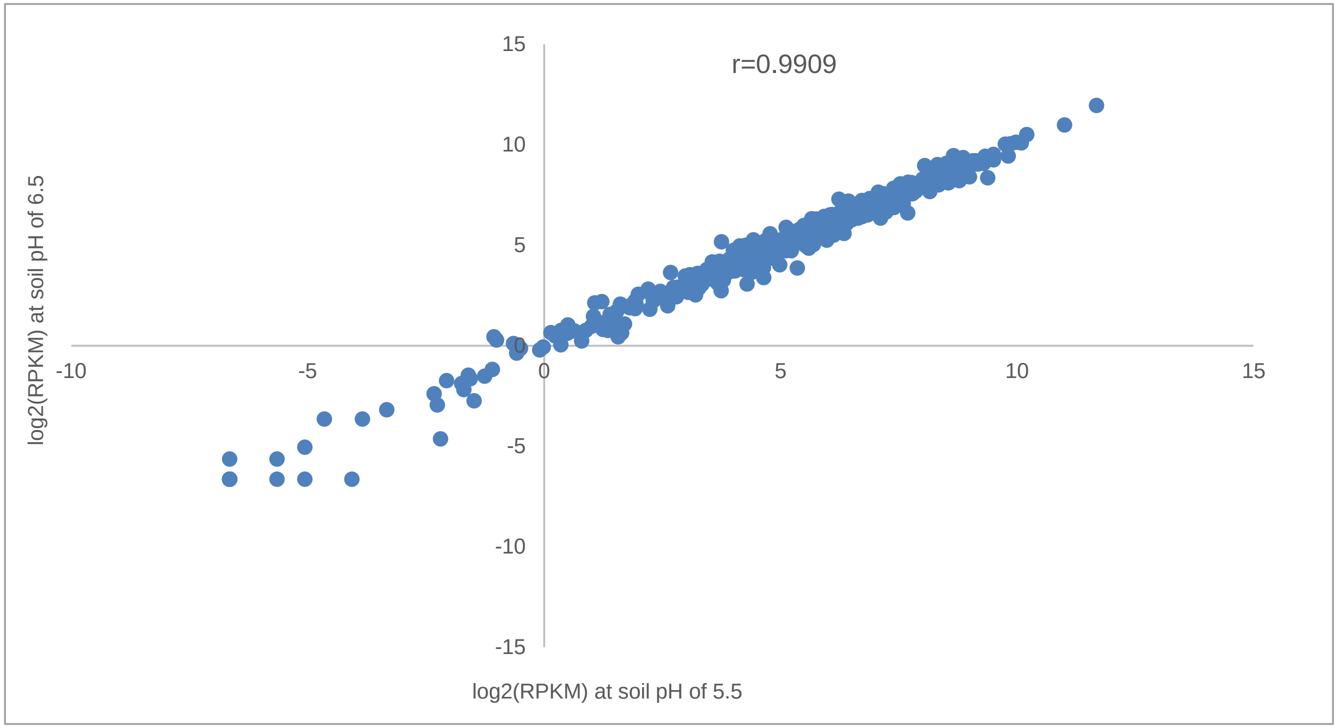

Figure S4. Scatter plot of expression levels in source tissues at two different soil conditions (pH of 5.5 and 6.5) of genes whose mRNAs were detected to transmit in only one of the two soil conditions.
